# Painting by Lesions: Functional Networks Affected by White Matter Lesions Are Associated with Poorer Cognition

**DOI:** 10.1093/geroni/igaa057.1586

**Published:** 2020-12-16

**Authors:** Rachel Crockett, Chun Liang Hsu, Roger Tam, Todd Handy, Teresa Liu-Ambrose

**Affiliations:** 1 University of British Columbia, Vancouver, British Columbia, Canada; 2 Harvard Medical School, Boston, Massachusetts, United States

## Abstract

Cerebrovascular disease (CvD) is the second most common cause of dementia. Its associated pathology, such as white matter lesions (WML), is associated with reduced cognition. Due to the high variability, the relevance of WML location remains unknown. We hypothesised that although the location of WMLs may appear sporadic, they may actually lie within common functional networks. We used novel imaging methods to map the location of WMLs in a clinical sample with the functional connectivity associated with the same location in the human connectome. This identified the functional networks containing the largest WML load (>50%) in older adults with CvD. We then analyzed the association between level of disruption to these networks and measures of global cognition and executive functions. Included in this study were 164 older adults (>55 years old) with CvD. Cognition was assessed using the: 1) Montreal Cognitive Assessment (MoCA); 2) Stroop Colour Word Test; 3) Trail Making Tests; and 4) Digit Symbol Substitution Test. Our results found that the visual network and ventral attention network (VAN) surpassed the 50% overlap threshold with 85% and 66% overlap respectively. Additionally, after controlling for multiple comparisons and age, the level of disruption to the VAN was significantly associated with poorer global cognition, as measured by the MoCA (p=.001). These novel findings identify the functional networks most affected by the presence of WMLs in older adults with CvD and suggest that the disruption to the VAN caused by WML load may underlie the deficits seen in cognition in this population.

